# Advances in prognostic models for osteosarcoma risk

**DOI:** 10.1016/j.heliyon.2024.e28493

**Published:** 2024-03-26

**Authors:** Yi Yao, Dapeng Wang, Li Zheng, Jinmin Zhao, Manli Tan

**Affiliations:** a Guangxi Engineering Center in Biomedical Materials for Tissue and Organ Regeneration, The First Affiliated Hospital of Guangxi Medical University, Guangxi Medical University, Nanning, 530021, China; b Collaborative Innovation Centre of Regenerative Medicine and Medical Bioresource Development and Application Co-constructed by the Province and Ministry, The First Affiliated Hospital of Guangxi Medical University, Guangxi Medical University, Nanning, 530021, China; c Department of Orthopedics, The First Affiliated Hospital of Guangxi Medical University, Nanning, 530021, China; d Life Sciences Institute, Guangxi Medical University, Nanning, 530021, China

**Keywords:** Osteosarcoma, Risk-prognosis model

## Abstract

The risk prognosis model is a statistical model that uses a set of features to predict whether an individual will develop a specific disease or clinical outcome. It can be used in clinical practice to stratify disease severity and assess risk or prognosis. With the advancement of large-scale second-generation sequencing technology, along Prognosis models for osteosarcoma are increasingly being developed as large-scale second-generation sequencing technology advances and clinical and biological data becomes more abundant. This expansion greatly increases the number of prognostic models and candidate genes suitable for clinical use. This article will present the predictive effects and reliability of various prognosis models, serving as a reference for their evaluation and application.

## 1 Introduction

Predictive modeling, particularly regression modeling, has always been used in medical research to predict the progression and outcomes of various diseases [1]. With the emergence of next-generation sequencing technologies in osteosarcoma treatment, these new techniques have the potential to improve the precision and effectiveness of osteosarcoma therapy. Sequencing-based targeted treatment strategies are expected to improve outcomes for osteosarcoma patients [2]. Primary bone cancer is extremely rare, accounting for fewer than 1% of all newly diagnosed cancers. For example, in 2018, the United States diagnosed about 3450 new cases [3]. Metastatic (or secondary) bone cancer refers to cancer that spreads bone from other parts of the body, such as breast cancer that metastasizes to bone. Adults are more likely to develop cancerous tumors that metastasize to the bone than primary bone cancers. Consider the United States: as of the end of 2008, there were an estimated 280000 adults aged 18–64 had metastatic bone cancer [4]. As a result, different types of bone cancer must be distinguished. Osteosarcoma is a type of primary bone cancer caused by bone-forming cells known as osteoblasts in osteoid tissue (immature bone tissue), which typically occurs in the legs near the shoulders, arms, and knees of children and adolescents [5]. Osteosarcoma is a multifactorial disease with intricate interactions between various factors and mechanisms. When these factors and mechanisms interact, they cause dysregulation of cellular signaling pathways, resulting in disruption of bone tissue homeostasis. Because the majority of osteosarcoma tumors arise from the bone growth plate, disruption of precursor cell differentiation contributes to their development. Another important consideration is the functional changes in tumor suppressor genes and/or oncogenes. Furthermore, environmental factors that trigger epigenetic mechanisms can resulting in tumor suppressor genes and other genes expression disorder, including oncogenes. These occurrences have an impact on gene activation and silencing and are frequently observed in tumor-derived cells. Epigenetic processes such as DNA methylation, histone modification, nucleosome remodeling, and non-coding RNA involvement are frequently linked to the progression of osteosarcoma [6]. Chemotherapy is a treatment method that significantly improves patient survival [7], with a survival rate of approximately 70%. Over the last 40 years, the survival rate of osteosarcoma patients has not improved significantly [8]. As a result, in the face of complex carcinogenic factors and no progress in treatment, an increasing number of researchers have conducted extensive risk predictions using sequencing technology and clinical outcomes to identify target molecules associated with mortality.

In this review, we introduced some of the most recent developments in osteosarcoma predictive models, focusing on their predictive effects and theoretical basis. The literature search was carried out using PubMed (www.ncbi.nlm.nih.gov/pubmed) with the search term "Risk prediction model of Osteosarcoma". Hundreds of articles were found in the surveyed database, but only those about risk models were chosen. We reviewed the literature and compiled data on 25 osteosarcoma risk prognostic models, as shown in Table 1.

**Table 1:** Information on 25 prognostic models of osteosarcoma risk.

Sequence	Model Category	Samples used for training model	Genes		Area under the curve of ROC(AUC)				PMID	Common datasets (Sequence)
			Protective Genes (HR < 1)	Risk Genes (HR > 1)	1 year	2 years	3 years	5 years		
1	Hypoxia genes	TARGET database: 84 patients with osteosarcoma (RNA-seq)	GFPT2, ACTG2	CYFIP2, RASGRP2, DKK1, DLX2, KCNJ3, CHVP4C, KLK1, NRXN1, ABCA4, CORT	0.749	–	0.786	0.797	36686667	DLX2 in 1 and 15
2	Metabolic genes	Undefined: 131 patients with osteosarcoma	NUDT7, ALDH1A1, HNMT,	GLO1, SQLE, PAICS, PPAT, SLC19A1, FASN, AK2	0.81	–	0.85	0.9	35096022	
3	Glycolytic pathway genes	TARGET database: 87 patients with osteosarcoma (RNA-seq)	ADH5, DCN, G6PD, PGAM1, SDC3, PFKFB2, GNPDA2, HS2ST1	ZNF292, CDK1, INSR, FAM162A, GLCE	0.959	–	0.899	0.895	36387908	INSR in 3 and 18; DCN in 3 and 21; G6PD in 3 and 9
4	Glutamine metabolism-related genes	GSE21257: 53 patients with osteosarcoma Beadchip	ARL14, CEACAM21, ANXA10, TGFB2, NIN, MAN1C1, HOXD11, ZNF467	ALDOC，CLGN，KRT14，TCF，UBE2O，NFKBIB，TRAP1，RGSL1	–	–	0.900	0.920	35136353	TGFB2 in 4 and 14
5	Fatty acid and lactate metabolism–related genes	TARGET database: 84 patients with osteosarcoma (RNA-seq)	SLC7A7, ACSS2	MYC	0.841	–	0.750	0.750	36119478	SLC7A7 in 5 and 22; MYC in 5 9, 10 and 15
6	Energy metabolism-related genes	TARGET database: 76 patients with osteosarcoma (RNA-seq)	SLC18B1, RBMXL1, ATP6V0D1, HS3ST2, DOK3	CCAR1, C1QTNF1	0.770	–	0.920	0.910	32581649	
7	GPCR-related genes	TARGET database: 85 patients with osteosarcoma (RNA-seq)	CCR4, HCRTR2, DRD2, HTR1A, GPR3, GPR158	HTR1E, OPN3, GRM4, GPR144	–	0.966	0.923	0.939	35463319	
8	Immune-related genes	TARGET database: 84 patients with osteosarcoma (RNA-seq)	GJA5, APBB1IP, NPC2	FKBP11	–	0.960	0.930	0.890	33425993	
9	Ferroptosis‐related gene	TARGET database: 93 patients with osteosarcoma (RNA-seq)	ATG7, DPP4, EGLN1, G6PD, PEBP1, PGD, SLC39A8, SOCS1	ALOX15B, CBS, MUC1, MYC	0.806	–	0.818	0.838	34506683	G6PD in 3 and 9; MYC in 5 9, 10 and 15
10	Epigenetic modifications-related genes	TARGET database: 88 patients with osteosarcoma (RNA-seq)	EIF4E3	MYC, TERT, RBM34	0.861	–	0.772	0.771	34853590	EIF4E3, TERT, RBM34 in 10 and 20; MYC in 5 9, 10 and 15
11	Pyroptosis-related genes	TARGET database + GSE21257: 69 patients with osteosarcoma (RNA-seq) + beadchip	CPB1, CATSPER1, CD79A	CORT, ARMC4	0.826	–	0.806	0.787	36389801	
12	Autophagy-related genes	TARGET database: 93 patients with osteosarcoma (RNA-seq)	VPS18, AMBRA1, CDK5, MAPKAP1, ARL8B, TBC1D14, USP10, AKT1S1	BNIP3, SAFB2, PTPRS, LGALS8	0.779	–	0.814	0.865	34194501	
13	Necroptosis-Related lncRNAs	TARGET database: 85 patients with osteosarcoma (RNA-seq)	AL 354919.2	AP000851.2, AL391121.1	0.806	–	0.728	0.731	35692813	
14	Platelet-related genes	TARGET database: 85 patients with osteosarcoma (RNA-seq)	MAPK1, GNG12, KIF21B, ANXA5, GAS6, TGFB2	–	0.630	–	0.740	0.7200	36212395	GNG12 in 14 and 18; TGFB2 in 4 and 14
15	Cellular Senescence-Related Genes	TARGET database: 86 patients with osteosarcoma (RNA-seq)	EPHA3, LIMK1	MYC, DLX2	0.930	–	0.770	0.800	36106335	DLX2 in 1 and 15; MYC in 5 9, 10 and 15
16	Metastasis-related gene	TARGET database: 41 patients with osteosarcoma (RNA-seq)	CTGF, AMIGO2, EXOSC5	ABCA3, PREB, FHIT	1.000	1.000	0.969	–	34368151	
17	Ubiquitin-related gene	TARGET database: 84 patients with osteosarcoma (RNA-seq)	WDR53, FBXL5, UBE2L3	CORO6, DCAF8, UHRF2	0.957	–	0.890	0.919	36060009	
18	RAS-Related Gene	TARGET database: 84 patients with osteosarcoma (RNA-seq)	GNG12, EGFR, EFNA5, RALB, HGF, PRKACB, RAB5C	CALML3, CALML5, INSR, RRAS2, EFNA1, GNG4, IGF1R	0.772	0.917	0.837	–	36052285	GNG12 in 14 and 18; INSR in 3 and 18
19	Transcription Co-Factor–Related Gene	TARGET database: 93 patients with osteosarcoma (RNA-seq)	LMO2, MAML3, SIRT1	MTF2, RBPMS	0.607	–	0.713	0.736	35734428	
20	RNA Binding Protein	TARGET database: 84 osteosarcoma patients (RNA-seq)	TDRD6, TLR8, NXT2, EIF4E3, RPS27L	CPEB3, RBM34, TERT, RPS29, ZC3HAV1	0.840	–	0.870	0.880	34926575	EIF4E3, TERT, RBM34 in 10 and 20;
21	Osteoblast differentiation-related genes	TARGET database: 84 patients with osteosarcoma (RNA-seq)	TPM1, LOXL1, PSMD10, PEF1, TUBB, TUBA1A, DCN	S100A13, ST3GAL4, SERPINE2, FAM207A	0.834	–	0.792	0.796	35300639	DCN in 3 and 21;
22	Circular RNA-Related CeRNA	TARGET database: 44 patients with osteosarcoma (RNA-seq)	MLLT11, SLC7A7, PARVA	TNFRSF11B	0.867	–	0.955	0.934	34629900	SLC7A7 in 5 and 22
23	DNA methylation gene sites	TCGA dataset: 47 patients with osteosarcoma	cg04533248, cg12401425, cg13997435, cg15075357	–	–	–	–	0.861	35117331	
24	Super Enhancer Associated Genes	GSE39058: 41 patients with osteosarcoma (BeadChip)	ZP3, AMN1	LIMS1, SPARC, SAMD4A	0.895	–	0.845	0.813	33195283	
25	Pseudogene	TARGET database: 94 patients with osteosarcoma (RNA-seq)	RPL11-551L14.1, Rpl7ap28, RP11-326A19.5	Rp4-706a16.3	–	–	0.885	0.878	31146489	

## 2 Risk prognosis model based on hypoxia-related genes

A large body of evidence shows that tumor hypoxia has a significant impact on a variety of cellular processes, including cell apoptosis, proliferation, angiogenesis, immune response, metabolism, genomic stability, and metastasis [9]. Hypoxia in osteosarcoma patients is associated with lower chemotherapy resistance and survival rates [10]. Hypoxia, in particular, can influence the tumor immune microenvironment by increasing innate immune cell recruitment, inhibiting adaptive immune cell differentiation, and reducing adaptive immune cell functionality [11]. Furthermore, experts place a high value on hypoxia, which is a typical characteristics of the tumor immune environment. Hypoxia in the tumor microenvironment is closely related to the rapid proliferation of cancer cells, excessive formation of fibrous matrix, and insufficient blood supply [10]. It is widely acknowledged that bones are susceptible to hypoxia, and numerous researches have found that hypoxic microenvironment of osteosarcoma not only boost tumor cell proliferation and metastasis, but enhances tumor drug resistance [12]. Hypoxia can upregulate the expression of hypoxia inducible factors (HIF), nucleolar and spindle-associated protein 1 (NUSAP1), and NADH dehydrogenase (ubiquinone) 1 alpha subcomplex 4-like 2 (NDUFA4L2), promoting of osteosarcoma cells survival, epithelial-mesenchymal transition, invasion and migration, [13,14]. HIF expression may facilitate tumor metastasis [15], possibly because HIF1 stumulates the expression of Vascular Endothelial Growth Factor-A (VEGF-A), which related to tumor angiogenesis [16]. However, in many tumor cells overexpressing VEGF, abnormal blood vessel formation often occurs [17]. As a result, tumor cells can easily spread through these abnormal blood vessels, which also contributor significantly to immune therapy and chemotherapy resistance [18]. In addition to its role in tumor migration and invasion, NUSAP1 can regulate cell cycle progression by promoting microtubule accumulation [19] and is linked to drug resistance [20]. NDUFA4L2 is a subunit of electron transfer chain complex I, and hypoxia can induce its overexpression and precisely regulate its activity. Knocking out NDUFA4L2 in cells and cultured under hypoxic conditions promotes mitochondrial ROS production, this showed that NDUFA4L2 resists oxidative stress damage by inhibiting ROS generation [21]. In osteosarcoma, imbalance of the redox system induced JNK activation and mTOR inhibition mediate apoptosis and autophagy in the cells [22]. This phenomenon highlights the relationship between mitochondrial genes and osteosarcoma progression. Studies have shown mitochondrial and genomic dysfunction is common in osteosarcoma cells, and low levels of DNA can increase tumor invasiveness [23].

Han Tao et al. conducted a consensus clustering analysis on the sample, utilizing the mRNA expression levels of 200 hypoxia-related genes. Their findings revealed that these genes were capable of categorizing the sample into two distinct subtypes, exhibiting a significant difference in overall survival rates between the two groups [24]. This suggests that there could be more hypoxia-related genes associated with survival in osteosarcoma patients. Following differential gene analysis of the two subtypes and univariate Cox regression analysis, then 23 genes related to survival were screened out. Among them, there are 4 protective genes (HR < 1) and 19 risk genes (HR > 1). Lastly, 12 genes were used as components of the prognosis model, including CYFIP2, RASGRP2, DKK1, DLX2, GFPT2, KCNJ3, ACTG2, CHMP4C, KLK1, NRXN1, ABCA4, and CORT. Two genes (GFPT2 and ACTG2) have HR < 1, suggesting a protective effect, while the remaining genes are risk factors. This model can exactly forecast the condition of patients. The area under the ROC curve (AUC) at 1, 3, and 5 years is 0.749, 0.786, and 0.797. The AUC measures prediction accuracy and ranges from 0.5 to 1, with a higher value indicating greater accuracy. This implies that the prognosis accuracy of this model is not perfect. Sunitinib reduces KCNJ3 expression and promotes osteosarcoma cells apoptosis, which is consistent with the analysis results. When investigating the role of model risk factors, it was found that CYFIP2 is widely ecognized as a p53-driven proapoptotic protein. In gastric cancer, CYFIP2 knockdown was found to promote proliferation and colony formation while inhibiting apoptosis of these cells [25], contradicting the analysis results. RASGRP2 activates Rap1, which inhibits cell apoptosis and reduces TNF-induced ROS production [26]. In an osteosarcoma mouse model, constitutive overexpression of DKK1 accelerated tumor growth rate and bone destruction [27]. DLX2 is a homeobox transcription factor that promotes cancer stemness, radiation resistance, EMT, tumor survival, and tumorigenicity [28]. CHMP4C, a pyroptosis-related gene, is overexpressed in patients with osteosarcoma and has a promoting effect on tumor progression [29]. KLK1 is a type of Kalinin genes that not only degrades the extracellular matrix, but also promotes further tumor proliferation, invasion, and worsening [30]. In Ewing's sarcoma, knocking down neuron-1 (NRXN1) reduces the number of living cells and globular formation, and high levels are associated with poor outcomes [31]. ABCA4 was highly expressed in patients who responded well to chemotherapy [32], and GFPT2 was positively associated with strong tumor aggressiveness, which contradicted the results of Han's study [33]. Aside from Han Tao's research, there is currently no evidence that GFPT2 acts a protective factor, and Han Tao's article does not include literature with consistent result. ACTG2 was significantly upregulated in invasive osteosarcoma cell lines [34], which appears to contradict to the conclusion. When we say that some studies contradict Han Tao's findings in osteosarcoma, it's because he did not provide research literature that supported his findings, but can provide some opposing literature.

Overall, the use of hypoxia-related genes in the prediction of osteosarcoma has some credibility, but some genes have inconsistent functions in different cancers, necessitating additional research to determine their reliability in osteosarcoma. Furthermore, the impact of hypoxia on osteosarcoma extends far beyond these contents. some hypoxia related genes have been linked to immune cells, inflammatory markers, and genes involved iron death [35].

## 3 Risk prognosis model based on metabolism-related genes

Glycolysis, lipid metabolism, oxidative phosphorylation and glutamine catabolism are all metabolic pathways of cells. Tumor cells provide ATP for tumor development by regulating metabolic pathways [36]. Zhongpei Zhu et al. identified 876 genes associated with metabolic function and prognosis in osteosarcoma patients [37]. Then, the first 10 metabolic genes related to survival were used to construct a prognostic model to predict the prognosis of patients. The area under the ROC curve (AUC) at 1, 3, and 5 years was 0.81, 0.85, and 0.9. The AUC for the authentication cohort was also greater than 0.8, indicating excellent prognostic accuracy.

The metabolic mode of tumor cells is predominantly glycolysis [38]. In cancer tissues, the rate-limiting enzymes involved in glycolysis are activated and overexpressed, including hexokinase (HK), phosphofructose kinase (PFK), glucose transporter protein (GLUT), pyruvate kinase (PK) and lactate dehydrogenase (LDH) [39]. Knocking down HK2 reduces aerobic glycolysis, increases apoptosis, indicated that HK2 regulates the glycolytic pathway to promote osteosarcoma growth [40]. Four distinct genes encode PFK-2/FBPase-2 [41]. By regulating the expression of PFKFB1, the expression of glycolytic interrelated proteins (LDHA, GLUT9), invasion markers, and cell cycle regulatory factors can be regulated, thereby controlling the progression of osteosarcoma [42]. PKLR and PKM encode pyruvate kinase, which is the final key enzyme in glycolysis. Notably, downregulation of the PKM2 subtype significantly reduces osteosarcoma cell's migratory capacity. In vivo studies have shown that silencing PKM2 can restrain further deterioration of osteosarcoma [43]. Knocking down GLUT1 significantly reduced osteosarcoma cell growth and invasion [44]. LDHB knockdown controls the progression, proliferation and invasive activity of osteosarcoma cells, and its expression is linked to tumor TNM staging, recurrence, and survival [45]. Of course, sugar metabolism is determined by more then just these few genes, including the signaling pathway genes that connect glycolysis. Cancer cells are distinguished by more than just Warburg metabolism or aerobic glycolysis when it comes to amino acid synthesis. Recent studies have revealed their reliance on amino acid metabolism. In addition to glucose, proliferating cells require significant amount of amino acids, which are essential for protein synthesis [46]. For example, specialized osteoblasts involved in bone formation require high levels of ATP production [47]. In the osteogenic process, glutamine acts as an auxiliary energy source for osteoblasts, undergoing oxidation in the TCA cycle to contribute to facilitate the production of ATP by preosteoblasts [48]. Arginine deprivation by polyethylene glycol arginine deaminase (ADI-PEG20) causes cell cycle arrest and dependence on autophagy. The combination of ADI-PEG20 and autophagy inhibitor chloroquine can induce to necrotic apoptosis and apoptotic cell death [49]. The proliferation of tumor cells rely on glutamine, and the mitochondrial enzyme glutaminase (GLS) catalyzes the conversion of glutamine into glutamate. GLS is a rate limiting enzyme, and the inhibitor CB-839 when combined with metformin, inhibits cell growth and metastasis [50]. Among other amino acids, osteosarcoma cells rely on methionine, which may help to maintain cysteine levels, because cysteine can control the synthesis of glutathione, which plays a vital role in maintaining the homeostasis of the intracellular environment [51]. The metabolism of branched chain amino acids is similarly closely linked with tumor progression. Knocking down ANGPTL4 in osteosarcoma cells causes an accumulation of branch chain amino acids, by activating the mTOR signaling pathway and promoting the development of osteosarcoma [52]. Because of the important of amino acids in cancer, pathways involving amino acid metabolism have been investigated. For example, activation of the serine biosynthesis pathway (SBP) has been connected with a poor prognosis in osteosarcoma [53].

Regarding the lipid metabolism. More and more evidence points out that lipid metabolism is critical in tumor progression, tumor metastasis, and drug resistance [[54], [55], [56]]. Researchers have found that genes related to lipid metabolism have great potential for predicting the prognosis of many tumors, such as osteosarcoma, hepatocellular carcinoma, ovarian cancer, diffuse glioblastoma, and lung adenocarcinoma. As a result, targeting lipid metabolism is known as an original idea to treat cancer [57]. In a lipid omics study comparing metastatic (143B) and non-metastatic (HOS) human osteosarcoma cells to normal fetal osteoblasts (FOB), 15 different lipid categories were found, including over 1000 different lipid classes such as phospholipids, sheath lipids, and ceramides, glycolipids, and cholesterol. Researchers have confirmed that diacylglycerol is overexpressed in metastatic osteosarcoma cells, and compared to normal lipids, they can prevent the malignant development of osteosarcoma cells by blocking the synthesis of diacylglycerol [58]. Sphingolipids' role in drug resistance, radiation therapy, and targeted therapy for different types of malignant tumors is currently being investigated by researchers [59]. GLB1, a sphingolipid metabolism-related gene, its overexpression can prevent the osteosarcoma cell's proliferation migration, and invasion [60].

Regarding the energy metabolism. As osteosarcoma progresses quickly, metabolites and amino acids in the glycolysis and tricarboxylic acid cycle (TCA) rise dramatically. This may be because cancer requires a lot of energy and changes the body's synthetic metabolism [61]. The loss of function or gene deletion of the retinoblastoma (RB1) gene is an major cause of osteosarcoma [62], and RB1 lacks inducible mitochondrial oxidative phosphorylation [63]. Therefore, we have concluded that energy metabolism is related to the progression of osteosarcoma.

### 3.1 Glycolysis-related genes

A prognostic model based on glycolysis-related genes. Wei Huang et al. [64] used the GSEA enrichment method to obtain a gene set related to glycolysis, and then screened out genes related to the prognosis of osteosarcoma from the gene set, establishing a prognosis model consisting of 13 genes that can significantly predict patients prognosis. The AUC values at 1, 3, and 5 years was 0.959, 0.899, and 0.895, indicating the model's notably high predictive accuracy. Genes with a hazard ratio (HR) greater than 1 (CDK1, INSR, FAM162A, GLCE, ZNF292) were supposed as risk genes, for the reson that genes with HR lower than 1 (G6PD, ADH5, GNPDA2, DCN, PFKFB2, PGAM1, HS2ST1, SDC3) were designated as guardian genes. Finally, seven prognostic genes-ADH5, FAM162A, HS2ST1, INSR, G6PD, GLCE, and SDC3-were found to have the strongest correlation with tumor metastasis. Western blot experiments confirmed the high expression of the INSR gene, which was found to be critical in the context of metastasis in osteosarcoma. The RNA interference test revealed that siRNA INSR transfection markably decreased the invasion rate of MG-63 cells. Alcohol dehydrogenase 5 (ADH5) is considered an important tumor suppressor in gastric cancer and non-small cell lung cancer, playing an anti-tumor role [65,66]. Core protein polysaccharides (DCN) are multifunctional extracellular matrix that can inhibit angiogenesis and non-metastasis of osteosarcoma [67,68]. G6PD is a critical enzyme in glucose metabolism, however, previous research has shown that it is involvement in the proliferation of osteosarcoma cells [69], contradicting Wei Huang's findings. Phosphoglyceric acid mutase-1 (PGAM1) catalyzes the conversion of 3-phosphoglyceric acid to 2-phosphoglyceric acid in the glycolysis pathway. It is upregulated in various cancers and promotes cell proliferation. It has been be connected with a poor prognosis in many cancers [[70], [71], [72]], which contradicts Huang Wei's findings. ZNF292 is known to act as a latent tumor suppressor in the GI tract, including gastric, liver, and colorectal cancer [73]. According to reports, some CDK1 inhibitors can cause cell cycle arrest and apoptosis in osteosarcoma cells, decreasing their survival rate [74]. These two genes have an inhibitory effect on tumors and are classified as risk genes in Wei Huang's model, which is a contradictory finding. Activation of PFKFB2 can promote the Warburg effect in osteosarcoma, thereby accelerating cancer progression [75], contradicting to Wei Huang's finding. Hypoxia has been shown that in studies to promote the expression of Syndecan-3 (SDC3) on macrophages, which is positively correlated with macrophage gene markers, resulting in a better overall survival rate for melanoma tumor patients [76]. HS2ST1 is markably up-regulated in breast cancer patients cause structural changes in heparan sulfate as well as changes in growth factors binding, weakening signals via MAPK and other pathways. The decrease in E-cadherin and epidermal growth factor receptor (EGFR) signal and expression is related to decreasing in breast cancer cells activity, adhesion, migration and invasion [77]. C-myc targets INSR and IGF1R, which promoting tumor occurrence and metastasis in TSCC via NF-κB pathway [78]. The risk of many genes in Wei Huang's model contradicts existing research results, raising questions about whether glycolytic-related genes have different functions in different cancers or whether their predictive accuracy of these genes is insufficient, necessitating further validation.

### 3.2 Amino acid metabolism related genes

A prognostic model based on amino acid metabolism-related genes. Lu Wan et al. [79] used the glutamine metabolism-related genes to build a prognostic model consisting with 16 genes. Each gene in the model had prognostic significance, and the model's predictive power was improved. The high-risk group had a significantly lower 5-year overall survival rate (11%) compared to the low-risk group (88%), p < 0.0001. The AUC at 3 and 5 years is 0.92 and 0.90, which shows that the model has great predictive ability. Among the 16 genes in this model, overexpression of 8 genes is contacted with good patient prognosis (ANXA10, ARL14, CEACAM21, HOXD11, ZNF467, MAN1C1, TGFB2, NIN), and high expression of 8 genes is associated with poor patient prognosis (ALDOC, CLGN, KRT14, TCF, UBE2O, NFKBIB, TRAP1, RGSL1). Among them, ANXA10 is considered a prognostic biomarker and inhibitory factor for hepatocellular carcinoma, it's upregulation suppressed cell proliferation and migration [80]. ARL14 regulates lung adenocarcinoma cell proliferation, migration, invasion, and cell cycle via the CIDEC/ERK/p38 signaling pathway [81]. CEACAM21, is one of the carcinoembryonic antigen family, is overexpressed in immunoactivity samples [82]. Overexpression of HOXD11 is relevant to the invasiveness of advanced tumors, as well as low survival rate. HOXD11 activates FN1 transcription via the FN1/MMP2/MMP9 pathway, degrades the extracellular matrix, and accelerates epithelial mesenchymal transition-like metastasis [83]. The high expression of ZNF467 is linked to shorter MFS in prostate cancer patients [84], which contradicts the research findings of Lu Wan et al. Overexpression of MAN1C1, inhibits colony formation, shortens the S phase of the cell cycle, and decreases migration ability [85]. Contrary to Lu Wan et al. findings, transforming growth factor-β2 causes malignant phenotypes in pluripotent proteoglycans and hyaluronic acid in human osteosarcoma cells [86], suggesting that this growth factor may be associated with these cells' capacity for metastatic. NIN expression increases during angiogenesis [87]. ALDOC can regulate cancer cell invasion [88]. CLGN is linked to aldosterone production in aldosterone-producing adenomas [89]. KRT14 deletion significantly reduces the migration, invasion and peritoneal metastasis in triple-negative breast cancer [90]. The TCF-1 gene is upgraded in osteosarcoma, and its knockout can cause cell cycle arrest, apoptosis, and DNA damage [91]. UBE2O deficiency can severely impair tumor initiation, growth, and metastasis while also inhibiting the metabolic reprogramming in tumor cells [92]. Downregulation of NFKBIB promotes cisplatin resistance in human gastric cancer [93]. TRAP-1 has anti-apoptotic properties and resists the toxicity of oxidants and anticancer drugs [94]. In testicular cancer, increased RGSL1 expression is linked to a higher patient survival rate [95]. To summarize, Lu Wan's prognostic model is highly accuracy, but there are still differences in gene functions and prognostic outcomes among different cancers, highlighting the need for more experiments to demonstrate molecular mechanisms or more clinical follow-up data to demonstrate the model's reliability.

### 3.3 Lipid metabolism related genes

A prognostic model constructed based on lipid metabolism-related genes. Zhou Wei Wu et al. constructed a prognostic model for osteosarcoma using genes related to fatty acid metabolism [96]. This model includes three genes: ACSS2, MYC, and SLC7A7. The survival analysis shows that the model can effectively predict patient prognosis, with an AUC value of 0.841, 0.750, and 0.750 at 1, 3, and 5 years. The HR of SLC7A7 and ACSS2 is less than 1, while the HR of MYC is greater than 1. The Western blot (WB) experiment revealed that the expression of ACSS2 and SLC7A7 were observably reduced in OS, whereas MYC expression increased significantly. There are reports that elevated SLC7A7 expression is connected with a poor prognosis in glioblastoma patients [97]. Furthermore, SLC7A7 is significantly upregulated in chemotherapy resistant ovarian cancer [98], contradicting the findings of Zhouwei Wu et al. According to some studies, activating ACSS2 can prevent calcium activated breast cancer from further worsening [99]. ACSS2 is up-regulated in human pancreatic ductal adenocarcinoma cell lines, and shRNA knockdown of ACSS2 inhibits cancer cell death while increasing cell cloning and the decreasing of intracellular pH, indicating that it is an active regulator for the pancreatic ductal adenocarcinoma treatment [100]. MYC is considered a driving gene in osteosarcoma [101]. In summary, the genes in this model contradict previous research findings, and further exploration is essential to determine the causes of this phenomenon.

### 3.4 Energy metabolism related genes

A prognostic model based upon energy metabolism-related genes. Naiqiang Zhu et al. constructed a prognostic model for osteosarcoma using genes related to energy metabolism [102]. The model includes seven genes, with CCAR1 and C1QTNF1 increasing of CCAR1 and connected with an increased risk of osteosarcoma as risk factor, while increased expression of DOK3, RBMXL1, HS3ST2, SCL18B1, and ATP6VOD1 as protective factors. This model effectively predicts patient prognosis, with AUC values greater than 0.7, 0.9, and 0.9 at 1, 3, and 5 years, respectively. In terms of risk factors, CCAR1 expression is apparently upregulated in hepatocellular carcinoma, is positively related to cancer cell dryness, and is strongly associated with poor clinical outcomes [103]. C1q and TNF-related 1 (C1QTNF1, also known as CTRP1) expression is upregulated in metastatic cancer, high CTRP1 level is connected with poor prognosis, and CTRP1 knockout inhibits cell proliferation and invasion, and tumor growth [104]. In terms of protective factors, there are currently no reported functional associations between SLC18B1 and cancer. RBMXL1 encodes a splicing protein that inhibits tumor progression and facilitates apoptosis of gastric cancer cells [105]. Some studies have also shown that RBMX/L1 acts by directly regulating the CBX5 transcription [106], while the tyrosine kinase inhibitor resistant lung adenocarcinoma cell line [107] shows a decrease in CBX5 expression [108], suggesting that CBX5 plays a part in drug sensitivity and indirectly suggesting that RBMX/L1 may play a role in drug sensitivity. DOK3 gene as a lung tumor suppressor factor [109]. In breast cancer cells expressing HS3ST2, an imbalance of TCF4-regulated ion transporters and an increase of cytoplasmic acidification increased the cells’ chemosensitivity to doxorubicin and paclitaxel [110]. ATP6V0D1 is the d1 subunit of the vacuolar H^+^ ATPase, which is involved in the acidification of lysosomes etc. [111]. Overall, the prediction accuracy of this model is satisfactory, and the gene functions in the model are consistent with those studied by previous researchers.

## 4 Risk prognosis model based on GPCRs related genes

G protein-coupled receptors (GPCRs) have recently been shown to play a critical part in bone growth and disease, with 92 GPCRs being linked to bone dysfunction [112]. GPCR is one of the biggest transmembrane protein family, to participate in a variety of biological processes, including bone growth and remodeling [113,114], tumor growth and metastasis [115], and are considered excellent drug targets. However, GPCRs are rarely studied in osteosarcoma, so Manli Tan et al. [116] used the GPCRdb database to obtain 395 human non-olfactory GPCRs. Then osteosarcoma gene expression and clinical data were from the UCSC XENA platform. Finally, a prognosis model for osteosarcoma based 10 GPCRs was constructed, among them, GPR158, CCR4, DRD2, HCRTR2, HTR1A, and GPR3 with an HR < 1, while HTR1E, OPN3, GRM4, and GPR144 with an HR > 1. The low-risk group in this model has a significantly higher survival probability, with AUC values of 0.966, 0.923, and 0.939 at 2-year, 3-year, and 5-year, highlighting the excellent predictive accuracy of the model. In terms of guardian factors, CCR4 is expressed in a variety of T cell subtypes, involved effector CD8 T cells. Chemotaxis analysis has shown that CCL22 therapy recruits CCR4^+^CD8 T cells [117], which is consistent with the finding of elevated CCR4 expression in the low-risk group. In addition, the low-risk group has a relatively high infiltration rate of CD8 T cells, both of which are associated with longer overall survival. HCRTR2 expression is decreased in many tumors, and often frequently accompanied by promoter hypermethylation [118]. Orexin activates the orexin receptor HCRTR2, which induces cell apoptosis [119], indicating that HCRTR2 shares biological characteristics with tumor suppressor genes. Regarding GPR3, its agonist has capacity to render apoptosis of breast cancer cells caused by the toxicity of cationic drugs [120], which indicates tumor inhibition [121]. HTR1A can inhibit DNA synthesis when specific agonists are used [122], and it is upgraded in non-invasive cancer cell lines [123]. In esophageal squamous cell carcinoma, GPR158 has higher methylation levels than mucosal tissue [124], which can cause cell apoptosis and is linked to improved survival rates in glioma patients [125]. DRD2 is considered as a tumor suppressor factor [126]. Reports state that DRD2 on the cell membrane can inhibit tumor growth by downregulating eEF1A2 [127], whereas eEF1A2 increases osteosarcoma progression and degradation by triggering the Akt/mTOR signaling pathway [128], highlighting DRD2's anti-osteosarcoma effects. On the other hand, for risk factors, research has shown that GRM4 high expression is linked with higher mortality in osteosarcoma patients [129]. In lung adenocarcinoma and acral melanoma, lower survival rate is connected with OPN3 expression [130,131]. For the first time, GPR144 (ADGRD2) has been linked to the prognosis of osteosarcoma. Interestingly, GPR144 has a close evolutionary relationship with GPR133 [132]. In glioblastoma, GPR133 expression is negatively correlated with patient survival. HTR1E activation can control the secretion of cytokines IL-6 and CXCL8 [133], which plays an important part in mediating the interaction between osteosarcoma and the lung, a process critical for metastasis [134]. In glioblastoma multiforme, HTR1E is also considered a risk factor [135]. Overall, the gene functions in this model are broadly consistent with previous research, and the model's predictive accuracy is satisfactory.

## 5 Risk prognosis model based on immune-related genes

Immunotherapy has recently demonstrated promise in treating a range of cancers. However, its use in osteosarcoma remains unexplored. Some believe that osteosarcoma may be sensitive to immunotherapy. It is noteworthy that the proportion of CD8^+^ infiltrating lymphocytes in osteosarcoma is higher than that in other subtypes of sarcoma [136], and the degree of infiltration is positively correlated with survival rate [137]. The expression levels of complement proteins C1QA, C1QB, and C1QC are negatively correlated with osteosarcoma patient prognosis, and these three genes are associated with tumor immune infiltration [138] To determine immune-related genes that can forecast the prognosis of osteosarcoma, Mingde Cao et al. collected 864 immune-related genes from literature [139], and eventually constructed a prognosis model using four genes (APBB1IP, FKBP11, GJA5 and NPC2). It was established that higher FKBP11 expression was associated with increased risk (risk factors), whereas elevated expression of APBB1IP, NPC2, and GJA5 was associated with decreased risk. This model effectively predicts patient prognosis, with areas under the ROC curve (AUC) value of 0.96, 0.93, and 0.89 at 2, 3, and 5 years, indicating the model's excellent predictive accuracy. The function of the model gene indicates that FKBP11 facilitates cell progression in oral squamous cell carcinoma via p53-related pathways [140]. The exact role of GJA5 in tumor development is unknown, but it can facilitate the connection of blood vessel wall cells and is a constitutive vascular gap junction protein [141,142], suggesting a potential link to vascular permeability. APBB1IP is an important signal integration node during T cell activation, influencing a variety of innate and adaptive immunity functions [143]. NPC2 is downregulated in hepatocellular carcinoma. Furthermore, low NPC2 levels may indicate a passive prognosis and contribute to procession of hepatocellular carcinoma by modulating of the ERK1/2 pathway [144]. Taken together, the functionality of these four genes correspond to descriptions in existing literature, demonstrating the four-gene model's reliability and strong performance in predicting prognosis.

## 6 Risk prognosis model based on ferroptosis‐related genes

Ferroptosis is an iron-dependent cell death marked by abnormal iron metabolism and lipid peroxidation [145]. In contrast to apoptosis, which causes specific cell death events at predetermined time points, ferroptosis is primarily a mechanism for maintaining cell integrity [146]. Liu, Q et al. found that inducing ferroptosis boost the sensitivity of osteosarcoma cells to cisplatin [147], as a result, novel cancer treatment approaches based on ferroptosis show great promise to treat cancer patients who are resistant to traditional therapies, particularly those centered on apoptosis pathways [148].

Ting Lei et al. [149] obtained 112 ferroptosis-related genes (FRGs) from the GO and KEGG databases, and obtained 15 prognosis related FRGs through univariate COX regression analysis. After conducting LASSO regression analysis, 12 prognostic genes were verified for use in constructing a prognostic model. This model can significantly predict patient survival rate, with AUC of 0.806, 0.818, and 0.838 at 1, 3, and 5 years, indicating that the model's prediction accuracy is relatively high. The model's high expression of seven genes is related to higher survival rate, as protective factors, these genes are DPP4, ATG7, SOCS1, SLC39A8, PGD, PEBP1, and G6PD. The model's high expression of five genes is associated with a lower survival rate, as risk factor, these genes are CBS, ALOX15B, MYC, MUC1, and EGLN1. For the model's protective factors, diabetes patients treated with the DPP4 depressant sitagliptin after colorectal cancer surgery have a higher overall survival rate than patients treated with other diabetes medications [150]. DPP4 is overexpressed in thyroid cancer, and down-regulation inhibits cell growth and metastasis [151], indicating that DPP4 could be a risk factor, which contradicts Ting Lei's findings. ATG7 expression is associated with the regulation of oxidative stress in osteosarcoma. Silenced the ATG7 gene can boost osteosarcoma cell proliferation and migration while inhibiting oxidative stress and iron death [152]. SOCS1, a targeted regulatory factor for miR-331-3p, inhibits SOCS1 expression by binding to it's 3′UTR. Overexpression of miR-331-3p significantly inhibits osteosarcoma cell proliferation, metastasis, and invasion, suggesting that SOCS1 may be a risk factor, which contradicts Ding Lei's findings. SLC39A8 is a gene that encoding ZIP8 protein. ZIP8 controls cell sensitivity to the cisplatin, and overexpression of ZIP8 regulates cisplatin-induced cell apoptosis [153]. Silencing of PGD can inhibit cell proliferation, survival, and migration [154]. PEBP1 (RKIP) has been shown to be decreased in all kinds of cancers, and its absence is closely bond with cancer metastasis [155]. G6PD activation is a first-line response against oxidative stress in cancer cells, and is thought in association with immune escape, tumor progression, and chemotherapy resistance in many cancers [156], which is contradicts Ding Lei's findings. Highly expression of CBS accelerates colorectal adenocarcinoma cell proliferation, migration, and invasion [157]. ALOX15B is considered to have tumor-inhibitory effect in prostate cancer and breast cancer, but it's expression is associated with poor prognosis in colorectal cancer [158]. Myc is involved in osteosarcoma progression and accelerates cell invasion with the MEK-ERK pathway activation [101]. Abnormal glycosylation of MUC is associated with cell transformation from normal to malignant phenotypes in human cancer, thus, MUC1 is the primary target for cancer vaccine design and development [159]. EGLN1 accelerates the progression of nasopharyngeal carcinoma by promoting the further resistance of p53 ubiquitination in an hydroxylase dependent manner [160]. In summary, the prognostic model constructed with FRGs performs well in predicting patient prognosis, but the genes that comprise the model also have opposing prognostic functions in osteosarcoma and other cancers. This necessitates further investigation of the principle through experiments.

## 7 Risk prognosis model based on epigenetic modification gene

In recent years, researchers have proposed that m6A modification, as an epigenetic change, can regulate RNA splicing, translation, protein degradation and homeostasis, including methyltransferase (CBLL1, KIAA1429, METTL14, METTL3, RBM15, WTAP, RBM15B and ZC3H13). Demethylase (ALKBH5 and FTO) and m6A binding proteins (FMR1, IGF2BP1-3, LRPPRC, HNRNPA2B1, HNRNPC, ELAVL1, YTHDC1-2 and YTHDF1-3) [161]. Recently, Hanji Huang et al. discovered a strong link between RBM15 and the formation of osteosarcoma metastasis as well as reduced patient survival, suggesting that RBM15 may be a biomarker used to predict the prognosis of patients with osteosarcoma [162].

Siyu Liu et al. [163] Recently performed differential expression and survival analysis on epigenetic-related genes such as m6A, histone modification, RNA binding proteins, transcription factors and DNA methylation enzymes. Four independent prognostic genes were analyzed by multivariate Cox to build up a prognostic model: EIF4E3, RBM34, MYC, and TERT. The high-risk group in the model had a remarkably higher mortality, with AUC values of 0.861, 0.772, and 0.771 at 1, 3, and 5 years, indicating the model's good diagnostic performance. In this model, high expression of EIF4E3 is associated with low risk, while high expression of RBM34, MYC, and TERT is affiliated with high risk. According to research, EIF4E3 is a tumor suppressor by recognizing atypical patterns in methyl-7-guanosine caps [164]. RBM34 is overexpressed in hepatocellular carcinoma and is also bond with negative clinical pathology and prognosis [165]. Maintaining telomere length allows cells to avoid crises and attain immortality. In the majority of cancers, telomerase activation is used to maintain telomere length, a process that consists of two major components, one of which is the enzyme subunit known as telomerase reverse transcriptase (TERT) [166]. In summary, the model was reliable because the functions of the genes in it are largely consistent with descriptions in other literature.

## 8 Risk prognosis model based on pyroptosis-related genes

Pyroptosis is a kind of programmed cell death cause by inflammation, that distinguishes it from other programmed cell death mechanisms [167]. Numerous studies have implied that pyroptosis plays a key role in the pathogenesis and progression of various cancers. Nonetheless, the role of pyroptosis in cancer is complex, and it can have cancer-suppressive or cancer-promoting effects depending on the type of cancer [168]. Some researchers have pointed out that pyroptosis can provide a new treatment for osteosarcoma by changing the tumor immunosuppressive microenvironment [169]. Jun Han et al. [170] obtained the pyroptosis-related genes that are differentially expressed in osteosarcoma. Finally, using stepwise multivariate Cox regression, five genes were identified for the prognostic model: CORT, Cpb1, armc4, CatSper1 and CD79a. The AUC values of 1, 3, and 5 year were 0.730, 0.878, and 0.867. Among them, CORT and ARMC4 served as risk factors, while CPB1, CATSPER1 and CD79a served as protective factors. However CORT served as an inhibitor in breast tumorigenesis [171], ARMC4 is one of tumor suppressor genes that negatively regulates NF-κB in colorectal cancer. Overexpression of ARMC4 can inhibit the growth and invasion of tumor cells by controlling the NF-κB pathway [172]. Functionally, CATSPER1 facilitates the progression of colorectal cancer. CATSPER1 can activate the PI3K/AKT signaling pathway at the molecular level and promote the further progression of colorectal cancer [173], which contradicts Jun Han's. Findings. The overexpression of carboxypeptidase B1 (CPB1) is bound up with ductal carcinoma in situ and is bond with better survival outcomes [174], Consistent with Jun Han's findings. In triple-negative breast cancer, B-cell markers (CD79A) are enriched in patients with complete pathological response to neoadjuvant duvalizumab plus chemotherapy. This result tends to think that CD79A is a marker of B cell enrichment, which may be helpful for immunotherapy. It seems reasonable to regard it as a protective factor. In summary, the prognosis model constructed by pyroptosis-related genes has a good prediction function in osteosarcoma, but the function of some model's genes contradict the findings of other literature. Therefore, more examples are required to verify the model's accuracy.

## 9 Risk prognosis model based on autophagy-related genes

Autophagy has been described as a double-edged sword that leads to cancer, because it can not only remove the damaged substances in the human body in time to play an anti-tumor role, but also promote its further development after the occurrence of tumors [175]. In osteosarcoma cells, autophagy increases the proliferation and development, resists cancer treatment, and preserves the survival pathway of cancer stem cell pools within the tumor. However, which signal will promote the transition from survival autophagy to the process of death? Is an autophagy threshold required to cause cell death? To obtain answers to these questions may need to be risk scoring of all autophagy-related genes.

Qian H et al. [176] used univariate COX regression analysis to identify 12 autophagy-related genes with prognostic significance among 222 autophagy-related genes confirmed in previous studies, and then used them to construct a prognostic model. Among them, AKT1S1, ARL8B, AMBRA1, CDK5, MAPKAP1, USP10, TBC1D14, and VPS18 had HR values less than 1, indicating that they were protective factors, while BNIP3, SAFB2, PTPRS, and LGALS8 have HR values greater than 1 and were considered risk factors. This model has significant prognostic effects, with AUC values of 0.779, 0.814, and 0.865 at 1, 3, and 5-year, respectively. This indicates that the model has a good prediction accuracy. In terms of protective factors, up-regulation of VPS18 can promote tumor cell growth resistance to chemotherapy drugs, while silencing VPS18 gene can increase the resistance to DOX and DOC chemotherapy drugs [177], which seems to contradict the view as a protective factor. AMBRA1 acts as a tumor suppressor in mouse model [178], and it's absence promotes the growth and invasion of melanoma [179]. Previous studies have linked CDK5 overexpression to lower survival rates in osteosarcoma patients, which contradicts the notion that it is a protective factor [180]. Upregulation of MAPKAP1 is closely related to the progression of cervical squamous cell carcinoma [181], contradicting its role as a protective factor. ARL8B Knockdown can reduce lysosomal exocytosis and radiation survival cells' invasiveness. The overexpression of the ARL8B gene is significantly accompanied by the poor prognosis of breast cancer patients [182], contradicting the notion that ARL8B is a protective factor. AKT1S1 encodes PRAS40, which has been referred to as a key downstream target of Ewing's sarcoma protein (EWS). Knocking down PRAS40 inhibits Ewing's sarcoma cell proliferation and metastatic growth [183]. In summary, the function of autophagy-related genes in osteosarcoma is different from other cancers. Therefore, it is recommended usng this type of gene as a prognostic model for osteosarcoma, which requires more detailed mechanism research to evaluate the applicability of this model.

## 10 Risk prognosis model based on necroptosis-related lncRNAs

Long non-coding RNAs (lncRNAs) have been discovered and characterized over the last decade, and they play a key role in the progression of various disease. According to reports, lncRNAs derived from intron transcripts [184] were discovered to be differentially expressed in osteosarcoma, and their magical function of encoding micropeptides was revealed. Furthermore, natural antisense RNAs are part of the lncRNA family and are able to stimulate, decrease, or entirely silence gene expression of sensory transcripts. Experiments have revealed that these lncRNAs are differentially expressed in osteosarcoma [185]. The single-cell atlas suggests that some lncRNAs may play a key part in the immune system because they are highly expressed in myoid cells [186]. Many lncRNA play a crucial part in the malignancy and chemotherapy effects of osteosarcoma [187]. The relationship between lncRNAs and osteosarcoma survival rate is an important research topic, but it is rarely reported comprehensively.

Wang et al. identified 243 prognosis-related lncRNAs and constructed a prognostic model with Necroptosis related lncRNAs [188]. The model includes three lncRNAs, with AL 354919.2 acting as a protective factor and AL391121.1 and AP000851.2 as risk factors. The high-risk group in the model had a high mortality, with an AUC value of 0.808, 0.728, and 0.731 at 1, 3, and 5 years, indicating the model's good diagnostic performance. Other studies have shown that AL 354919.2 acts as a protective factor in bladder cancer [189], which is in accordance with Wang's finding. AL391121.1 is overexpressed in renal clear cell carcinoma patients with favourable prognosis [190], which contradicts to the predictive effect found by Wang et al., AP000851.2 acts as a protective factor in breast cancer [191], which contradicts the predictive effect found by Wang et al. Prognostic models based on lncRNA are currently in the exploratory stage, but they may provide inspiration for future research. However, there is seldom study on the molecular mechanisms of lncRNAs of osteosarcoma. Their prognostic effects in different tumors were inconsistent, indicating that clinical prediction remains a significant challenge.

## 11 Prognostic model constructed by other factors

In addition to the aforementioned factors used to construct prognosis models, there are platelet-related genes [192], cell aging-related genes [193], transfer-related genes [194], ubiquitin-related genes [195], RAS pathway-related genes [196], transcription cofactor related genes [197], RNA binding proteins [198], osteoclast differentiation-related genes [199], and cyclic RNA related CeRNA [200], DNA methylation related genes [201], super-enhancer related genes [202], and pseudogenes [203].

## 12 Discussion

To summary, the credibility of the constructed prognostic model is largely determined by the number of samples used during computation. Although there are inconsistencies between the clinical statistical data of some models currently in development and some experimental data from the past, it is difficult to determine which is more effective in guiding clinical practice. Because statistics and experimental research are both sciences, when their conclusions differ, it is worth considering carefully whether to criticize each other for being correct or incorrect or to continue investigating the underlying principles of their conclusions. Among the various prognostic factors mentioned above, some are intersected rather than classified separately. For example, many hypoxia-related genes are actually metabolism-related genes, which may also involve immune-related genes. Using them for classify and include prognosis models is the result of scientific division of labor and fine differentiation. Many scientists can only focus on research in one academic area. Of course, some researchers are not limited to a specific type of gene, but have identified 217 differentially expressed genes associated with overall survival by distinguishing patient outcomes (alive or death) [204]. When clinical big data and second-generation sequencing data arrive in large quantities, everyone wants to know if previous molecular biology research can withstand clinical statistics. Undoubtedly, the individual of life is complex, and many genes are also spatiotemporal regulated. The functions displayed in one organ may not necessarily be mirrored in another. To understand the role of these genes, it is necessary to investigate not only their functions in a specific disease but also to explore more general molecular mechanisms. Similarly, the findings derived from statistical results should be considered at multiple levels, such as whether the cardinality is large enough, the expression changes of a specific gene in cancer, and how to determine whether it is an oncogene or a "passenger" gene.

With the development of technology, single-cell technology provides a more refined composition of the tumor microenvironment, which helps to understand the cellular origin of prognostic genes [205]. Yun Liu et al. [206] linked 85 differentially expressed and prognostic genes to the single-cell atlas of osteosarcoma, finding that nearly half of these samples were relatively overexpressed in two subgroups of osteoblasts. Because osteosarcoma originates from osteoblasts [5], it is feasible to identify specific cell subpopulations based on biomarkers and predict patient survival rates. This finding suggests that by combining single-cell technology and survival analysis, we can identify key cell subpopulation in osteosarcoma, provide insights for histology scoring, precise treatment, and diagnosis, and develop diagnostic methods for determining tumor malignancy by identifying cell subpopulation biomarker. In addition, associating immune-related prognostic genes with a single-cell atlas [207] would be a valuable study because once we know which immune cell subpopulations the prognostic genes originate from, corresponding immunotherapies can be developed, which will directly benefit patient survival rates.

As shown in Table 1, the samples for the osteosarcoma prognostic model are mostly from those with gene expression profiles in the TARGET osteosarcoma database. Therefore, it is important to discuss whether these samples are suitable for statistical patient survival rates. As is known to all, metastasis is a priori knowledge of the increased death of osteosarcoma [208]. In these TARGET osteosarcoma samples with gene expression profiles, metastasis also resulted in lower survival rate [116]. Because these TARGET osteosarcoma samples conform to prior knowledge, we believe they are suitable for the prognostic analysis.

## 13 Summary and outlook

Clinical prognosis research is an important method for identifying treatment targets and developing detection kits. The development of clinical prognosis models has revealed that multiple factors influence osteosarcoma prognosis. Currently, multiple preliminary verifications have been performed, providing an important foundation for the selection of new treatment plans to improve prognoses. However, these models have not yet been widely applied in clinical diagnosis and treatment, reminding us that good prognostic models have promising clinical application prospects. In addition, there is also a problem with limited sample size when constructing prognostic models. In the future, it will be necessary to collaborate with multiple centers to collect more clinical sample data for prognostic analysis.

## Ethics approval and consent to participate

Not applicable.

## Funding statement

This study was supported by the Guangxi Scientific Research and Technological Development Foundation (Grant No. GuikeAB21220062 and GuikeABAB23026049), the 10.13039/501100001809National Natural Science Foundation of China (grant number 82360426), and the Foundation of the Collaborative Innovation Centre of Regenerative Medicine and Medical Bioresource Development and Application Co-constructed by the Province and Ministry (Grant No: CICRMMBR-TC-2023011).

## Data availability statement

This article is a review article and hence no data was used for the research described in the article.

## CRediT authorship contribution statement

**Yi Yao:** Writing – original draft, Investigation. **Dapeng Wang:** Writing – original draft, Investigation. **Li Zheng:** Writing – review & editing, Supervision, Conceptualization. **Jinmin Zhao:** Supervision, Methodology, Conceptualization. **Manli Tan:** Writing – review & editing, Validation, Investigation, Conceptualization.

## Declaration of competing interest

The authors declare that they have no known competing financial interests or personal relationships that could have appeared to influence the work reported in this paper.
